# Chronic lymphocytic leukemia (CLL) with Reed–Sternberg-like cells vs Classic Hodgkin lymphoma transformation of CLL: does this distinction matter?

**DOI:** 10.1038/s41408-022-00616-6

**Published:** 2022-01-28

**Authors:** Rebecca L. King, Alia Gupta, Paul J. Kurtin, Wei Ding, Timothy G. Call, Kari G. Rabe, Saad S. Kenderian, Jose F. Leis, Yucai Wang, Susan M. Schwager, Susan L. Slager, Neil. E. Kay, Amber Koehler, Stephen M. Ansell, David J. Inwards, Thomas M. Habermann, Min Shi, Curtis A. Hanson, Matthew T. Howard, Sameer A. Parikh

**Affiliations:** 1 Division of Hematopathology, Mayo Clinic, Rochester, MN USA; 2 Division of Hematology, Mayo Clinic, Rochester, MN USA; 3 Department of Quantitative Health Sciences, Rochester, MN USA; 4 Department of Hematology/Oncology, Mayo Clinic, Scottsdale, AZ USA

**Keywords:** Chronic lymphocytic leukaemia, Hodgkin lymphoma

## Abstract

The distinction between chronic lymphocytic leukemia/small lymphocytic lymphoma (CLL/SLL) with isolated Hodgkin/Reed–Sternberg cells (CLL-HRS; background milieu with a paucity of inflammatory cells) and overt transformation to classic Hodgkin lymphoma (CLL-HL; mixed inflammatory background) is incompletely understood. This retrospective study examined the clinicopathologic features of CLL-HRS (*n* = 15) and CLL-HL (*n* = 31) patients seen over the past three decades from a single institution. The phenotypic features of Reed–Sternberg cells in both groups were similar, including expression of CD30, CD15, and PAX5, as well as EBV status. However, a spectrum of background CLL/SLL infiltration amongst the HRS cells was noted on pathologic review, and four patients had both diagnoses, either concurrently or in succession. The median overall survival (OS) of patients with CLL-HRS was 17.5 months compared to 33.5 months for patients with CLL-HL (*P* = 0.24). Among patients with CLL-HRS, those who received Hodgkin-directed therapy had a significantly longer median OS (57 months) compared to those who received CLL-directed therapy (8.4 months, *P* = 0.02). Our clinical and pathologic findings suggest a biologic continuum between CLL-HRS and CLL-HL and indicate that CLL-HRS patients may benefit from Hodgkin-directed therapy.

## Introduction

Progression of chronic lymphocytic leukemia/small lymphocytic lymphoma (CLL/SLL) to classic Hodgkin lymphoma (CHL) as a form of Richter transformation is an uncommon but well-documented event and occurs in >1% of CLL/SLL patients [1]. Sequencing studies of the immunoglobulin heavy chain variable region (*IGHV*) genes have proven clonal relationship between the CLL/SLL and Reed–Sternberg (HRS) cells in a subset of cases, supporting this as a true transformation phenomenon in some instances [2–7]. Generally, the pathologic diagnosis of CHL in this context is straightforward and requires HRS cells in a mixed inflammatory background similar to what is seen in *de novo* CHL [8].

In contrast, HRS cells can occur in a background of otherwise typical CLL/SLL without overt transformation to CHL, a phenomenon termed “CLL/SLL with HRS-like cells” (CLL-HRS). The World Health Organization (WHO) 2017 classification of lymphoid neoplasms emphasizes that the distinction between overt CHL transformation (CLL-HL) and CLL-HRS lies in the presence or absence of the mixed inflammatory background [8]. Although relatively few comprehensive studies of CLL-HRS exist, the emphasis has been on using histomorphology to distinguish this entity from CLL-HL since the phenotype of the HRS cells in both settings appear similar [7, 9]. Additionally, studies have shown that, as with CLL-HL, clonal relatedness can often be seen between the HRS cells of CLL-HRS and the CLL cells [3, 4, 7].

It is well established that CHL arising from CLL has an aggressive course with overall survival (OS) less than that of either CLL/SLL alone or de novo HL even when treated with standard HL-directed regimens [1, 9]. However, a recent multi-institution study reported improved outcomes of a contemporaneous cohort of patients with CLL-HL, where the median OS exceeded 13 years in patients treated with doxorubicin, bleomycin, vinblastine, and dacarbazine (ABVD)-based therapy [10]. In addition, the results of the German CLL study group support these findings [11]. Due to the rarity of CLL-HRS and the inevitable heterogeneity in its management, relatively few clinically well-documented series of CLL-HRS patients exist. Additionally, these patients may be excluded from a series of CLL-HL patients, leaving clinicians with little data on how these patients are managed. The largest retrospective series to date comparing cases of CLL-HRS (termed Type I pattern) to CLL-HL (termed Type II pattern) suggested that overall survival was similar for the two groups [7]. Thus, in spite of the pathologic emphasis on distinguishing the two, whether CLL-HRS clinically represents simply a spectrum of low-grade CLL/SLL, a transformation event similar to CLL-HL, or something in between, remains an unanswered question.

The goal of this retrospective study was to compare the clinicopathologic features of a cohort of CLL-HRS and CLL-HL patients with an emphasis on comparing the clinical outcomes of these entities. In addition, we sought to further define and delineate the pathologic features of CLL-HRS.

## Materials and methods

**Fig. 1:**
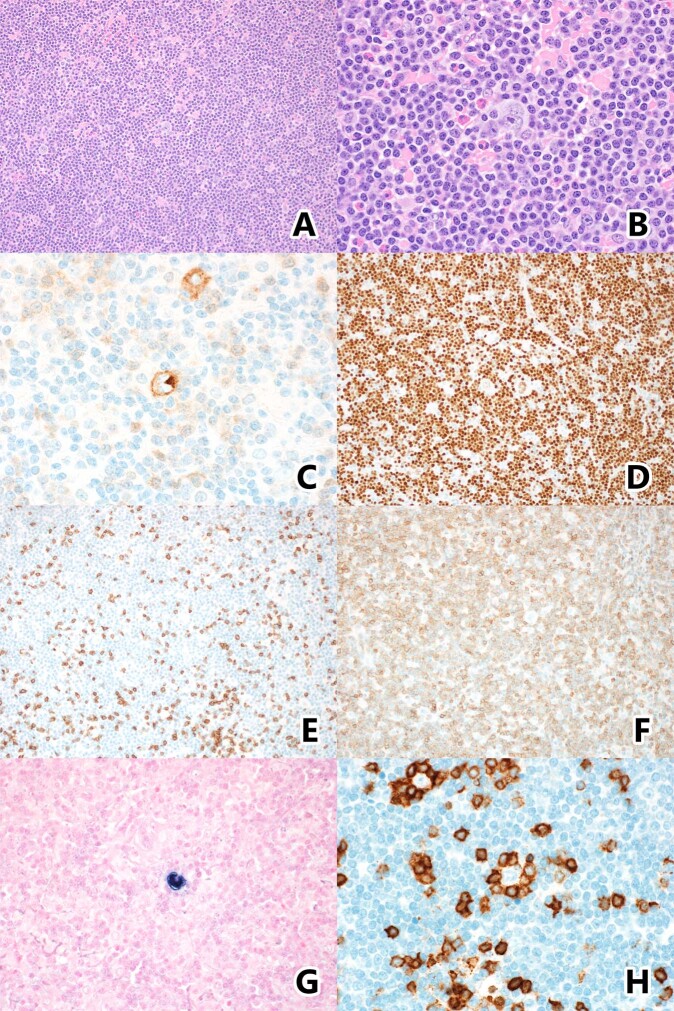
Typical case of CLL-HRS- illustrating HRS cells in a background of predominantly small CLL/SLL cells. Hematoxylin and Eosin 50x and 400x magnification (**A**, **B**). HRS cells express CD30 (**C**), and PAX5 (**D**) which also highlights the numerous small B cells. CD3 stains few admixed T cells (**E**), while CD5 stains the CLL B cells (**F**). EBER in situ hybridization stains the HRS cells (**G**). CD3 from another CLL-HRS case highlights prominent T cell rosettes around the HRS cells (**H**).

Following IRB approval, the Mayo Clinic CLL Database, Mayo Clinic Lymphoma Database, and Pathology archives were searched (1990-2020) for patients with a diagnosis of CLL/SLL and either classic Hodgkin lymphoma or CLL/SLL with Reed–Sternberg-like cells with prior informed consent to the utilization of their data for research purposes. The pathology materials were re-reviewed by three independent hematopathologists (RLK, AG, PJK). In the event of non-consensus on independent review, the cases were re-reviewed together and the consensus was able to be reached in all cases. Cases were classified using WHO 2017 criteria [8] as either CLL-HL (distinct areas including HRS cells in a mixed inflammatory background) or CLL-HRS (CLL with scattered HRS cells with minimal inflammatory background) (Fig. 1) [8, 12, 13]. Although a precise definition for “minimal inflammatory background” was not utilized, the CLL-HRS cases were considered as such if the predominant cells in the background were CLL/SLL. Epithelioid histiocytes and admixed reactive T cells were still accepted amidst the CLL/SLL background for a CLL-HRS diagnosis, as per prior studies [7].

CLL-HL cases were classified as such regardless of whether CLL/SLL was present at the time of the HL biopsy since all patients had a history of CLL/SLL. Immunohistochemical stains (IHC) for the following markers were performed via standard automated procedures (Benchmark XT, Ventana Medical Systems) to complete the panel if not already available in the archive: CD3 (Leica, NCL-L-CD3-565), CD20 (Dako, M0755), CD30 (Ventana, 790-4858), CD15 (Cell-Marque, CMC11521021), PAX5 (Dako, M7307), and CD45 (Dako, M0701). EBV-encoded RNA (EBER) in situ hybridization (ISH) (Ventana, 760-1209) was also performed on all cases in which material was available. Differences in stains by diagnosis type were calculated using Fisher’s exact or Chi-square tests as appropriate.

Clinical data including demographics, CLL-specific characteristics such as *IGHV* mutation status, genetic abnormalities detected by fluorescence in situ hybridization (FISH), therapy administered, and outcomes were obtained from the Mayo Clinic CLL Database. Time to diagnosis of CLL-HL and CLL-HRS was calculated from time of CLL diagnosis to the first biopsy specimen demonstrating either of these diagnoses. Line of therapy was calculated from the date of diagnosis of either CLL-HRS or CLL-HL. OS was calculated from the date of CLL-HL or CLL-HRS diagnosis to the date of death or last known alive date. OS was also calculated from the date of treatment initiation following CLL-HL or CLL-HRS diagnosis to the date of death or last known alive date. OS was plotted using the Kaplan–Meier method and was compared by diagnosis or by CLL- or HL-directed therapy via log-rank tests. Univariable Cox regression analyses were conducted to determine the impact of CLL-specific characteristics on OS. Additionally, we computed the Hasenclever index [14] on patients at the time of CLL-HRS and CLL-HL diagnosis and determined its impact on OS. Finally, we analyzed the impact of HL-directed treatment as a time-dependent covariate in a Cox regression analysis of OS from the date of CLL-HL or CLL-HRS. Statistical analyses were performed using SAS 9.4 (SAS Institute, Cary, NC).

## Results

### Patient cohort

**Fig. 2:**
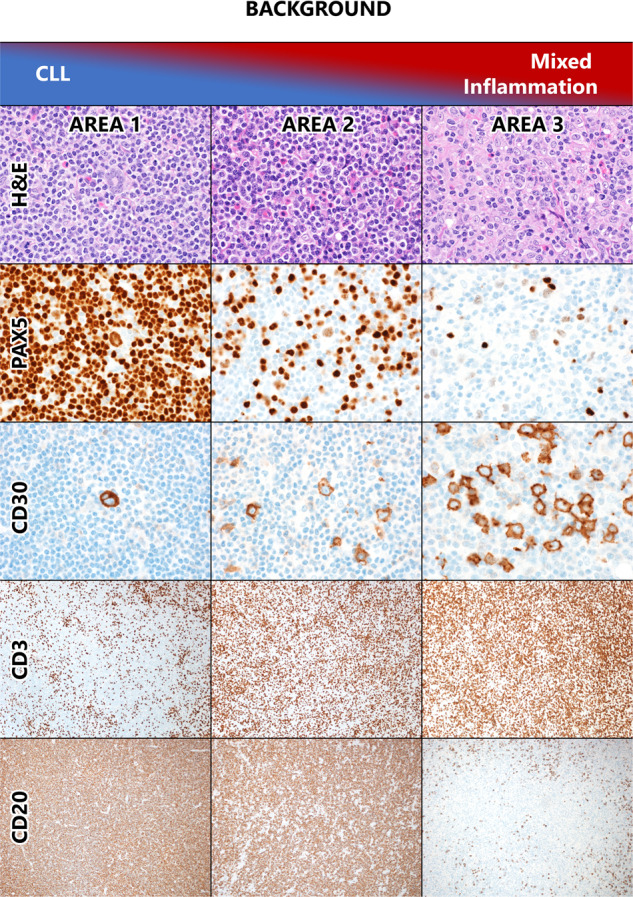
Case with areas of both CLL-HRS and CHL. The columns show three areas from a single lymph node in which areas of clear CLL-HRS (Area 1) and CLL-HL (Area 3) are present, along with intermediate areas in which the background is a mixture of CLL and inflammatory cells (Area 2). We hypothesize that CLL-HRS and CLL-HL exists on a biologic spectrum wherein HRS cells exist first in a cellular milieu composed of predominantly CLL cells (shown in blue in the top bar) and progress through stages where progressively more inflammatory cells (T cells, histiocytes, granulocytes, plasma cells) are recruited (shown in red in the top bar). In isolation, Area 2 would be diagnostic of CHL, while Area 1 would be diagnostic of CLL-HRS. Magnification: HE, PAX5, CD30 all 600x oil; CD3 and CD20 100x.

A total of 51 patients with CLL/SLL and either CLL-HRS or CLL-HL were identified, of whom five patients were excluded. Exclusion was due to reclassification to a diagnosis other than CLL-HL or CLL-HRS (EBV-positive DLBCL[*n* = 3], CLL/SLL[*n* = 2] with increased CD20 positive large cells). Table 1 shows the baseline characteristics of 46 patients included in the study. Among these patients, 29 were diagnosed as CLL-HL,15 with CLL-HRS, and two patients had biopsies in which distinct areas of CLL-HRS and CLL-HL were present in the same tissue at initial diagnosis, as shown in Fig. 2. For the purposes of clinical analysis, these two are included in the CLL-HL cohort; for pathology analysis, they are included in both cohorts. The types of biopsy in each diagnostic category were as follows: CLL-HRS (eight excisional, three core needles, four bone marrow); CLL-HL (ten excisional, nine core needles, ten bone marrow); CLL-RS and CLL-HL (one excisional one core needle). The median age at the time of CLL-HL or CLL-HRS transformation diagnosis was 72 years, and there was a male predominance in both groups (71% in CLL-HL and 87% in CLL-HRS). The median time from CLL to CLL-HL transformation was 6.6 years and CLL to CLL-HRS was 4.9 years (*p* = 0.49).

**Table 1:** Patient characteristics.

	CLL-HRS	CLL-HL	Total
**N**	15	31	46
**Median [range] age at CLL diagnosis**	65 [44–93]	63 [42–82]	63 [42–93]
**Median [range] age at CLL-HRS/CLL-HL diagnosis**	72 [47–93]	71 [52–89]	72 [47–93]
**Sex, males**	13 (87%)	22 (71%)	35 (76%)
**Median [range] time from CLL diagnosis, years**	4.9 [0–34.5]	6.6 [0–24.5]	6.2 [0–34.5]
**Characteristics at the time of diagnosis of CLL**			
**Median [range] Beta-2 microglobulin**	2.4 [1.8–7.4]	4.0 [1.9-8.3]	2.9 [1.8-8.3]
Missing	6	21	27
**Rai Stage**			
0	4 (33%)	10 (40%)	14 (38%)
I–II	7 (58%)	15 (60%)	22 (59%)
III–IV	1 (8%)	0 (0%)	1 (3%)
Missing	3	6	9
**Unmutated IGHV genes**	8 (89%)	7 (70%)	15 (79%)
Missing	6	21	27
**FISH**			
17delp	0 (0%)	2 (14%)	2 (9%)
11delq	4 (44%)	3 (21%)	7 (30%)
Trisomy 12	3 (33%)	4 (29%)	7 (30%)
None detected	1 (11%)	4 (29%)	5 (22%)
13delq	1 (11%)	1 (7%)	2 (9%)
Missing	6	17	23
**CLL-IPI risk**			
Low	1 (14%)	1 (13%)	2 (13%)
Intermediate	2 (29%)	2 (25%)	4 (27%)
High	4 (57%)	3 (38%)	7 (47%)
Very High	0 (0%)	2 (25%)	2 (13%)
Missing	8	23	31
**Characteristics related to diagnosis of CLL-HRS/CLL-HL**			
**Hasenclever Index**			
0–2	2 (20%)	6 (24%)	8 (23%)
3	5 (50%)	3 (12%)	8 (23%)
4	3 (30%)	9 (36%)	12 (34%)
5–7	0 (0%)	7 (28%)	7 (20%)
Missing	5	6	11
**Median [range] number of CLL treatments prior to diagnosis of CLL-HRS and CLL-HL**	0 [0–8]	1 [0–8]	1 [0–8]
**Therapy of CLL-HRS and CLL-HL**			
None	2 (13%)	0 (0%)	2 (4%)
CLL-directed	7 (47%)	2 (7%)	9 (20%)
HL-directed	6 (40%)	28 (93%)	34 (76%)
Missing	0	1	1

### Histopathology findings

Per the WHO definition [8], CLL-HL cases had a background of mixed inflammation and were distinct from areas of CLL/SLL, although some minimal infiltration of CLL cells into the HL background was accepted for this diagnosis. 19/31 (61%) of CLL-HL patients had residual CLL/SLL in the same biopsy as the CHL. In the majority of CLL-HL cases [24/31 (77%)], the inflammatory background in HL included a mixture of histiocytes, lymphocytes, and plasma cells, without eosinophils. Compared to *de novo* CHL, cases with eosinophils were less frequently seen as part of the inflammatory milieu [7/31 (23%)]. Fibrosis was a prominent feature in 16/31 (52%) cases. Necrosis was rare [2/31 (6%)].

Among the CLL-HRS cohort, which by definition had a minimal inflammatory background compared to CLL-HL cases, CD3 staining highlighted distinct rosetting of T cells around the HRS cells in 9/16 (56%) cases (Fig. 1H). 2/17 (12%) cases also contained increased prolymphocytes and paraimmunoblasts, forming prominent proliferation centers. Two cases demonstrated prominent clusters of epithelioid histiocytes amidst an otherwise monotonous CLL/SLL background. In CLL-HRS, eosinophils admixed with the background CLL cells and HRS cells were uncommon, seen in only one case.

Among the entire cohort, 15/46 (33%) had multiple biopsies (for suspicion of progressive disease) after the index diagnosis of either CLL-HRS or CLL-HL (not including biopsies showing only CLL/SLL or which were negative for lymphoma). As mentioned above, two patients had both CLL-HRS and CLL-HL seen in different areas of the same biopsy (Fig. 2). One of these with an initial diagnosis of CLL-HRS had a biopsy showing CLL-HL 19 months later. The other patient had CLL-HL and subsequently had a biopsy showing CLL-HRS after 32 months. One patient with CLL-HRS developed an EBV-positive DLBCL 3 months later. The histology of the lymph node specimens from the remaining 12 patients with multiple biopsies was concordant with their initial diagnosis (either CLL-HRS or CLL-HL).

### Immunophenotyping

The HRS cell phenotype in the two groups was similar (Table 2). In all but one case of CHL, the HRS cells expressed CD30 and PAX5 and were CD45-negative. CD20 expression in HRS cells was significantly more common in the CLL-HRS group than the CLL-HL group. HRS cells were EBER-positive in 81% of CLL-HRS and 63% of CLL-HL cases (*p* = 0.31). Although each case was classified as CLL-HL or CLL-HRS, the variable CD3-positive T-cell infiltrate (including the aforementioned CD3-positive T-cell rosettes) in cases of CLL-HRS and the frequent infiltration of CLL/SLL cells into the background of CLL-HL most resembles a spectrum of pathology rather than two discrete entities. This is exemplified in the two cases where both ends of the pathologic “spectrum” were present, i.e., in which areas diagnostic of CLL-HRS were seen in the same tissue as areas of overt CHL. As illustrated in Fig. 2, these cases also showed intermediate areas (Area 2) in which there was more mixed inflammation and less CLL/SLL than the CLL-HRS area (Area 1), but less mixed inflammation and more CLL/SLL than the CLL-HL areas (Area 3).

**Table 2:** Immunophenotyping in CLL-HRS compared to CLL-HL.

Stain	CLL-HRS*	CLL-HL*	*p*-value
	Positive/total(%)	Positive/total (%)	
**EBER-ISH**	13/16 (81)	17/27 (63)	0.31
**CD30**	16/16 (100)	30/31 (97)	1.0
**CD15**	8/16 (50)	24/31 (77)	0.06**
**CD20**	7/17 (41)	2/29 (7)	0.01
**CD45**	0/15 (0)	0/31 (0)	1.0
**PAX5**	16/16 (100)	26/28 (93)	0.53

*CLL-HRS* Chronic lymphocytic leukemia/small lymphocytic lymphoma with Hodgkin/Reed–Sternberg-like cells, *CLL-HL* Classic Hodgkin lymphoma, *EBER* Epstein Barr Virus encoded RNA, *ISH* in situ hybridization.

*Cases with both CLL-HRS and CLL-HL areas are included in both totals.

**Most *p*-values based on Fisher’s exact test; one based on Chi-square test.

### Treatment and outcomes of CLL-HRS and CLL-HL

**Fig. 3:**
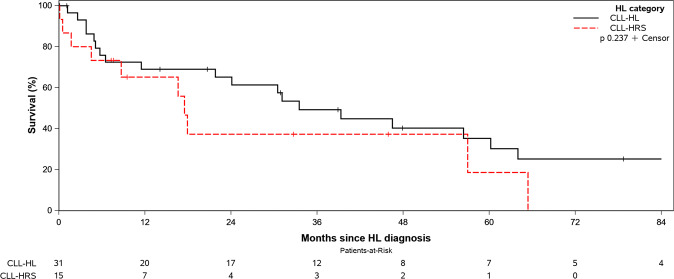
Overall Survival of all patients in the study, according to a diagnosis of CLL with Reed–Sternberg-like cells (CLL-HRS) and CLL-Hodgkin lymphoma (CLL-HL).

The median OS for the entire study cohort was 31.1 months. Although the median OS was shorter in CLL-HRS patients (17.5 months) compared to patients with CLL-HL (33.5 months), this difference was not statistically significant (*p* = 0.24, Fig. 3).

**Fig. 4:**
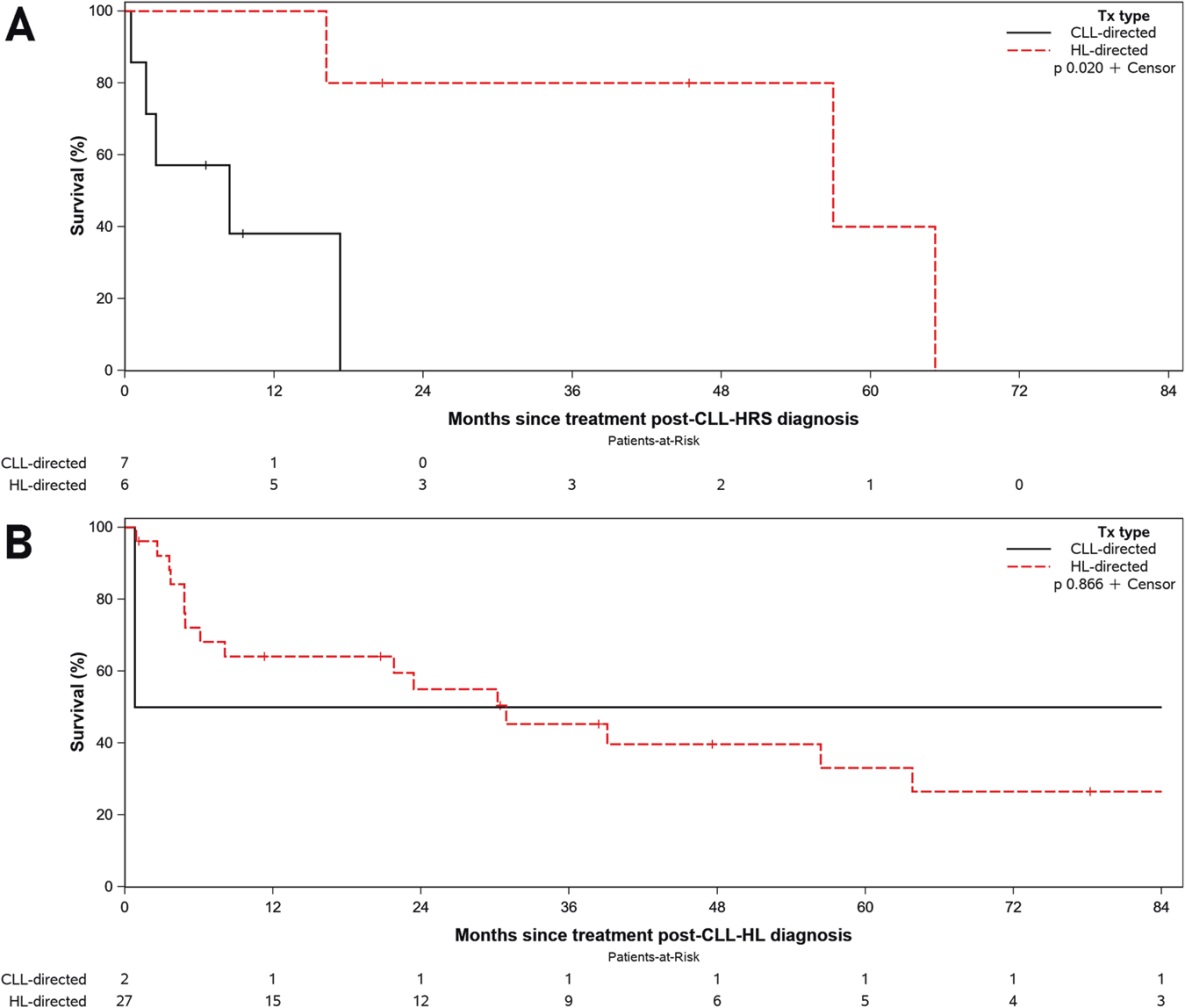
Overall survival based on treatment administered. **A** Overall survival among patients with CLL with Reed–Sternberg-like cells (CLL-HRS) according to the type of treatment administered —CLL-directed and Hodgkin lymphoma (HL)-directed therapy. **B** Overall survival among patients with CLL with Hodgkin lymphoma according to the type of treatment administered—CLL-directed and Hodgkin lymphoma (HL)-directed therapy.

Of the 13 CLL-HRS patients who were treated (Supplemental Table 1), seven (54%) received CLL-directed therapy as first-line treatment (four patients received rituximab with or without corticosteroids, two patients received chemoimmunotherapy, and one patient received acalabrutinib), and six (46%) received HL-directed first-line therapy (four patients received ABVD-based treatment, one patient received radiotherapy, and one patient received BCVPP (carmustine, cyclophosphamide, vinblastine, procarbazine, and prednisone). No significant clinical differences were identified to account for the difference in choice of therapy, although the sample size is small). Two patients did not receive any therapy. Patients who received initial CLL-directed treatment did not receive any HL-directed therapy during their clinical course. On the other hand, of the six CLL-HRS patients who received first-line HL-directed therapy, four received salvage HL-directed therapy (including one autologous stem cell transplantation), and one patient received therapy for DLBCL for what was presumed at the time to represent progression to DLBCL. In addition, 3/6 patients also required subsequent therapy for recurrent CLL. The median OS from treatment among patients with CLL-HRS was significantly longer with first-line CHL-directed therapy (*n* = 6; 57.0 months) compared to those patients who received first-line CLL-based therapy (*n* = 7; 8.4 months) (*p* = 0.02, Fig. 4a).

Among 31 CLL-HL patients who were treated (Supplemental Table 1), first-line treatment consisted of ABVD-based therapy (one patient received rituximab plus AVD) in 18 (62%) patients; seven patients (22%) received non-ABVD-based therapy (three patients received BCVPP, three received ChlVPP [chlorambucil, vinblastine, procarbazine, and prednisolone] and one received MOPP [mechlorethamine hydrochloride, vincristine, procarbazine, and prednisone]); one patient (7%) received brentuximab vedotin; one (7%) patient received brentuximab vedotin and nivolumab; one (7%) patient received radiotherapy; two patients received CLL-directed treatment (combination of anti-CD20 monoclonal antibody and corticosteroids). The treatment status of one patient was unknown. Of the 28 patients who received first-line HL-directed therapy, seven required salvage therapy (three received HL-directed therapy, three received both CLL- and HL-directed therapy, and one patient received CLL-directed therapy). No patient underwent an autologous stem cell transplant.

Of the two patients who received first-line CLL-directed therapy, one patient received HL-directed therapy 3 weeks later, and one patient received no subsequent treatment. The median OS from treatment among patients with CLL-HL was not significantly different between those who received HL-directed therapy (*n* = 27; 30.9 months) compared to CLL-directed therapy (*n* = 2; 48.9 months; *p* = 0.87, Fig. 4b).

Univariable analysis of age, sex, CLL-HL vs. CLL-HRS diagnosis, Hasenclever index, and treatment status of CLL at the time of CLL-HL or CLL-HRS diagnosis did not impact OS; additionally, receipt of HL-directed therapy was not associated with OS (HR = 0.7; 95% CI 0.4–1.4; *p* = 0.35) (Supplemental Table 2).

## Discussion

The distinction between Hodgkin lymphoma Richter transformation in CLL/SLL and the rare but seemingly distinct phenomenon of CLL/SLL with HRS-like cells has been only rarely examined in the literature. Given the paucity of data, pathologists struggle with the morphologic “line” between CLL-HRS and CLL-HL, and clinicians struggle with whether to manage patients with a diagnosis of CLL-HRS as Hodgkin lymphoma or as CLL/SLL. This study presents detailed clinical and pathologic documentation of one of the largest cohorts of CLL-HRS patients in the literature and a concurrent cohort of CLL-HL patients, all from a single institution. Our findings suggest that clinically and pathologically, these patients show a spectrum of findings, and these two entities likely exist on a biologic continuum. Furthermore, our findings suggest that CLL-HRS patients managed with Hodgkin-directed therapy, rather than CLL-directed therapy, may have superior outcomes.

The question of whether CLL-HRS represents simply a morphologic variant of CLL/SLL or a true form of CHL has been debated in the literature. Our results confirm previous reports that the phenotype of HRS cells in CLL-HRS is similar to those in CLL-HL and *de novo* CHL [4, 7]. In addition, both show frequent EBV expression in HRS cells (60–80%) which is in line with a prior series from the National Institutes of Health (NIH) which showed positivity in 71% of cases with no difference between the two groups [7]. Our results suggest that CD20 is more frequently expressed in CLL-HRS than CLL-HL. This is likely due to our exclusion of cases with the constellation of Hodgkin-like morphology, EBV positivity, and strong-diffuse CD20 as these cases are best classified as EBV-positive diffuse large B cell lymphoma. In general, when present, CD20 expression within the CLL-HRS cases is weak and variable, although cases with stronger expression can be seen and do not preclude the diagnosis when CD30 is also strong in the HRS-like cells. One interesting case of CLL/SLL with scattered large CD20 positive/CD30 negative/EBV negative cells with an immunoblastic (rather than HRS-like) cytology was excluded and not considered to be within the spectrum of CLL-HRS for the purposes of this study.

The WHO 2017 devotes relatively little space to the distinction between CLL-HRS and CLL-HL, emphasizing that the distinction lies in the presence of the mixed inflammatory background or a CLL/SLL background [8]. Review of our cases, however, supports a pathologic spectrum between the two entities rather than a discrete break. We considered whether smaller biopsies led to a diagnosis of CLL-HRS more frequently since perhaps overt CHL was missed in those cases, however, our data did not show such a trend and in fact, the CLL-HRS cases were enriched in excisional biopsies compared with the CLL-HL cases. Regardless, excisional biopsy should be encouraged in this setting if possible, as both diagnoses can be challenging on small specimens. It is well known that in a subset of cases of CLL-HL, the patient will have residual CLL/SLL in the background [1, 6, 7, 9, 12, 15, 16]. Typically, there is a clear delineation between the two morphologies, allowing for a confident diagnosis of CHL. However, in some cases, there is a degree of CLL/SLL infiltration into the background of otherwise typical CHL.

Conversely, two cases of otherwise typical CLL-HRS in our study showed prominent clusters of epithelioid histiocytes populating the background CLL/SLL, and one CLL-HRS case showed an infiltrate of eosinophils. Additionally, once a CD3 stain is applied, the distinction between CLL-HRS and CLL-HL is further obscured because reactive T cells, not readily apparent on H&E, are often prominent in the background of CLL/SLL, and in many cases form rosettes around the HRS cells. As such, it can be challenging for pathologists to determine precisely how much inflammatory background is acceptable for CLL-HRS. These results are virtually identical to what is described in the NIH series. They paradoxically support both the ability of independent pathologists to apply similar criteria for CLL-HRS vs. CLL-HL and highlight the pathologic spectrum from CLL-HRS at one end to CLL-HL at the other.

Lending further support to the inherent relationship of the two, we had two cases with distinct areas of CLL-HRS and overt CHL in the same biopsy and two additional patients who had both CLL-HRS and CHL at different timepoints. Although we did not undertake clonality studies, several prior reports have confirmed that through *IGHV* sequencing of microdissected HRS cells, a subset of both CLL-HRS and CLL-HL cases can be proven to be clonally related to the underlying CLL/SLL [2–4, 7, 12]. In the NIH series, 29% (4/14) and 53% (10/19) of CLL-HRS and CLL-HL cases showed clonal relationship, and no clinical differences were noted between the related and unrelated cases [7]. In routine clinical practice, microdissection of HRS cells is not practical, and all cases of CLL-HL are considered to represent Richter transformation.

One of the most significant findings of this study is the observed difference in OS in CLL-HRS patients who were treated with CLL-directed therapies compared to HL-directed therapies. Given the retrospective nature of this study, these data must be interpreted with caution. However, these findings have important implications for a scenario in which clinical guidelines are lacking and suggest that hematologists treating patients with CLL-HRS should consider HL-directed therapy.

Our study has several strengths. All pathology slides were reviewed by three pathologists who were unaware of the clinical outcomes of these patients. We performed a comprehensive assessment of the histopathologic and phenotypic findings and correlated them to the clinical outcomes of our patients using the Mayo Clinic CLL Database [17, 18]. Our study has a few limitations as well. It is a single-center retrospective study, and results should be validated in additional patient cohorts. Patients enrolled in this study were diagnosed with CLL-HRS and CLL-HL over the course of the last three decades where substantial improvements have occurred in the management of patients with CLL and HL.

In summary, the results of this retrospective study indicate that, despite the absence of a well-developed inflammatory milieu in patients with CLL-HRS, the clinical characteristics and many histopathologic findings are similar to patients with CLL-HL. The inferior outcome of CLL-HRS patients treated with CLL-directed treatments underscores the importance of accurately identifying CLL-HRS and treating these patients with HL-directed therapies.

## Supplementary information

Supplemental Tables
