# Supplementary material for: Chronic lymphocytic leukemia (CLL) with Reed–Sternberg-like cells vs Classic Hodgkin lymphoma transformation of CLL: does this distinction matter?
Source: Blood Cancer J. 2022 Jan 28;12(1):18. doi: 10.1038/s41408-022-00616-6 (PMC8799721; doi:10.1038/s41408-022-00616-6)
Supplement: Supplementary file 1 — Supplemental Tables [file 41408_2022_616_MOESM1_ESM.docx]

**Supplemental Table 1: Types of first-line treatment administered to patients with CLL-HRS and CLL-HL**

|  | **Hodgkin-directed therapy** | | | | **CLL-directed therapy** | | |
| --- | --- | --- | --- | --- | --- | --- | --- |
|  | *ABVD-based treatment* | *Non-ABVD based chemotherapy* | *Radiation therapy* | *Brentuximab-vedotin based therapy* | *Anti-CD20 monoclonal antibody +/- steroids* | *CIT* | *Acalabrutinib* |
| **CLL-HRS (n=15)*** | 4 (31) | 1 (8) | 1 (8) | 0 | 4 (31) | 2 (15) | 1 (8) |
| **CLL-HL (n=31)*** | 18 (60) | 7 (23) | 1 (3) | 2 (7) | 2 (7) | 0 | 0 |

*two patients with CLL-HRS did not receive any treatment; and the treatment status of 1 patient with CLL-HL was not known

Abbreviations used: ABVD: doxorubicin, bleomycin, vinblastine and dacarbazine; CIT: chemoimmunotherapy.

**Supplemental Table 2: Univariable analysis of factors associated with overall survival after a diagnosis of CLL-HL or CLL-HRS**

| **Variable** | | **Hazard Ratio (95% CI)** | **p-value** |
| --- | --- | --- | --- |
| Age at CLL diagnosis | | 1.01 (0.97-1.05) | 0.63 |
| Age at CLL-HL or CLL-HRS diagnosis | | 1.02 (0.98-1.06) | 0.33 |
| Male sex | | 1.93 (0.83-4.5) | 0.13 |
| CLL-HRS vs. CLL-HL | | 1.59 (0.73-3.46) | 0.24 |
| Hasenclever score | <3 | 1.07 (0.34-3.35) | 0.91 |
|  | 4 | 2.55 (0.92-7.08) | 0.07 |
|  | 5-7 | 1.57 (0.47-5.28) | 0.46 |
| Prior CLL therapy | | 1.59 (0.76-3.29) | 0.22 |
| HL-directed vs not HL-directed therapy (time-dependent) | | 0.7 (0.4-1.1) | 0.35 |
